# PD50 Does The New Healthcare Reform Improve Job Satisfaction Among Village Clinic Doctors In China? A Meta-Analysis

**DOI:** 10.1017/S026646232400309X

**Published:** 2025-01-07

**Authors:** Lifang Zhou, Yuncong Yu, Junping Liu, Jiaxian Shao, Wenqiang Yin, Qianqian Yu, Zhongming Chen

## Abstract

**Introduction:**

Since 2009, the Chinese government has launched a new health system reform that affected primary healthcare significantly. We aimed to analyze the factors associated with job satisfaction among village clinic doctors since the new healthcare reform, and to provide a reference for the next stage of reform.

**Methods:**

We systematically searched one English (PubMed) and two Chinese literature databases (CNKI and Wanfang Data). Cross-sectional studies containing information related to job satisfaction among village clinic doctors in China were included. The total job satisfaction among village clinic doctors was estimated using a random effects meta-analysis. Differences in study-level characteristics among groups were estimated using subgroup analysis and meta-regression.

**Results:**

We identified 17 cross-sectional studies investigating a total of 28,468 village clinic doctors in China. The pooled job satisfaction value was 0.40 (95% confidence interval [CI]: 0.32, 0.49). The results showed that lower job satisfaction was reported in the period from 2016 to 2020 (0.33, 95% CI: 0.23, 0.42) than in the period from 2010 to 2015 (0.51, 95% CI: 0.33, 0.70). The main factors influencing job satisfaction among village clinic doctors were salary (odds ration [OR] 1.71, 95% CI: 1.23, 2.36), number of training sessions (OR 2.56, 95% CI: 1.68, 3.90), age (OR 3.45, 95% CI: 2.22, 5.35), and level of education (OR 0.68, 95% CI: 0.40, 1.15).

**Conclusions:**

Since the new health system reform, only 40 percent of village clinic doctors in China are satisfied with their work and it is likely this figure will continue to decrease. Those with higher salaries, more training sessions, and greater age had higher job satisfaction. In contrast, village clinic doctors with a higher level of education had lower job satisfaction.

